# Pharmacological investigations of newly synthesized oxazolones and imidazolones as COX-2 inhibitors

**DOI:** 10.1016/j.jsps.2024.102191

**Published:** 2024-10-19

**Authors:** Iqra Saleem Naz Babari, Muhammad Islam, Hamid Saeed, Humaira Nadeem, Hassaan Anwer Rathore

**Affiliations:** a Punjab University College of Pharmacy, University of the Punjab, Lahore, Pakistan; b Riphah Institute of Pharmaceutical Sciences, Riphah University Islamabad, Pakistan; c College of Pharmacy, QU Health, Qatar University, Doha 2713, Qatar

**Keywords:** Oxazole, Imidazoles, COX-2 inhibitors, Carrageenan induced paw edema, Tissue antioxidant activity, H& E staining, ELISA, PCR

## Abstract

Oxazoles and Imidazoles are heterocyclic compounds with significant biological activities. The present study explores the pharmacological effects of some new oxazole and imidazole derivatives as potential COX-2 inhibitors. Docking studies of the compounds against targeted proteins COX-2 and TACE manifested good binding affinities for both the targets supporting their anti-inflammatory potential. Compounds (3F-A, 3F-B, N-A, N-B) were evaluated for *in vivo* anti-inflammatory effects by carrageenan-induced paw edema. Among all, compound N-A was found to be the most effective as it displayed most pronounced reduction in inflammation that was comparable to indomethacin. The *in vivo* tissue antioxidant activity was performed for estimation of the level of catalase, GSH, GST, and thiobarbituric acid in paw tissue. The results exhibited that targeted compounds improved the oxidative stress and restored the expression of enzymes. H &E staining revealed that aforesaid compounds displayed well-defined restoration of cellular damage. Compound NA exhibited maximum structural and functional preservation. Reduction in the expression of inflammatory markers was also analyzed by ELISA and maximum reduction in protein expression (COX-2 and TNF-a) was observed for compound N-B. Quantification of mRNA was done using PCR and a decrease in the expression of COX-2 mRNA level in treatment groups was depicted by all the new compounds but N-B showed maximum reduction in enzyme expression. All the results obtained from the present study have shown the significant anti-inflammatory potential of new compounds via the COX-2 inhibition pathway.

## 1 Introduction

Inflammation is an adaptive response of the immune system by which the detrimental stimuli impasses by host resulting in the healing of the damaged tissue. It is a protective mechanism in response to unfavorable effects such as tissue injuries, microbial infection, and other virulent conditions. The classical symptoms of inflammation include pain, swelling, heat, and redness (Chopra et al., 2024). Inflammatory diseases are characterized by abnormal inflammatory responses as a major manifestation. The inflammatory mechanism is supported by a large number of mediators that form a complicated regulatory system therefore general inflammatory process comprises stimulators, sensors, mediators, and effectors with each component deciding the type of inflammatory response. In acute inflammation various molecular and cellular events remarkably reduce the impending injury, this mitigation process helps in the restoration of tissue homeostasis however if acute inflammation becomes uncontrolled it would lead to chronic inflammation resulting in various chronic inflammatory diseases (Qazi et al., 2022). There are many pathways of inflammation and compounds having anti-inflammatory potential may specifically inhibit one out of many pathways or several pathways to produce their anti-inflammatory effects (Ansari et al., 2021).

Today a large number of drugs involve heterocyclic systems showing effective therapeutic effects. The incorporation of the heterocyclic system in the structure of compounds or drugs imparts significant specificities in their biological responses. Heterocyclic compounds are cyclic compounds, organic having at least one hetero atom like oxygen, nitrogen, Sulphur, and some other atoms in their rings. Nowadays, heterocyclic compounds like Oxazoles, pyroles, Imidazoles, Thiazoles, etc. are used in many biological fields because of their measurable biological effects on various diseases (Al-Mulla, 2017). New scientific insights, discoveries, and applications are taking place related to new drugs containing heterocyclic rings and more than 90 % of the current drugs possess heterocyclic rings as functional therapeutic moieties (Saini et al., 2013).

Oxazolones (compounds containing a five-membered heterocyclic ring with oxygen and nitrogen) (Joshi et al., 2017) and imidazolones (compounds containing a five-membered heterocyclic ring with two nitrogen atoms at a distance of one carbon atom) (Abdellatif and Fadaly, 2017) are reported to have anti-inflammatory effects by various mechanisms. Most commonly they act by interfering with reactive oxygen species by selectively inhibiting inflammatory mediators like COX1 or COX2 (Mohamed et al., 2017) by inhibiting TNF-a production (Walser and Flynn, 1980, Laval et al., 2000) and by inhibiting tyrosinase activity (Samad and Hawaiz, 2019, Peng et al., 2022).

## 2 Materials

### 2.1 Chemicals and reagents

All the chemical reagents used in the current study were purchased from Merck, Sigma Aldrich, Macklin, and Daejung. TLC sheets were obtained from Merck to assess the progress of the reaction. The chromatograms were observed and analyzed by UV- lamp (Spectroline) under a short wavelength of 254 nm and a long wavelength of 365 nm. The melting points of all compounds were checked by a digital melting point apparatus (Stuart). The characterization of compounds was done by NMR (Bruker, 400MHZ) with TMS as an internal standard and FT-IR (Bruker OPUS 7.518) spectrophotometer. DMSO, formalin, normal saline, DTNB, carrageenan (CRG), and indomethacin were purchased from Sigma-Aldrich (Taufkirchen, Germany). Analytical-grade reagents were used without further purification.

### 2.2 Method

For synthesis of oxazolones and imidazolones general schemes described by Kumar et al. (2008) and El-Araby (2012) were followed with minor modifications. Some basic characterization is discussed in this article. Full-blown characterization and spectral analysis (Saleem Naz Babari, 2024) of compounds has been reported by the author previously. This article discusses brief and basic highlights of the synthesis of new compounds and the detailed pharmacological investigations of some selected compounds from the aforementioned new series as COX-2 inhibitors.

**Fig. 1:**
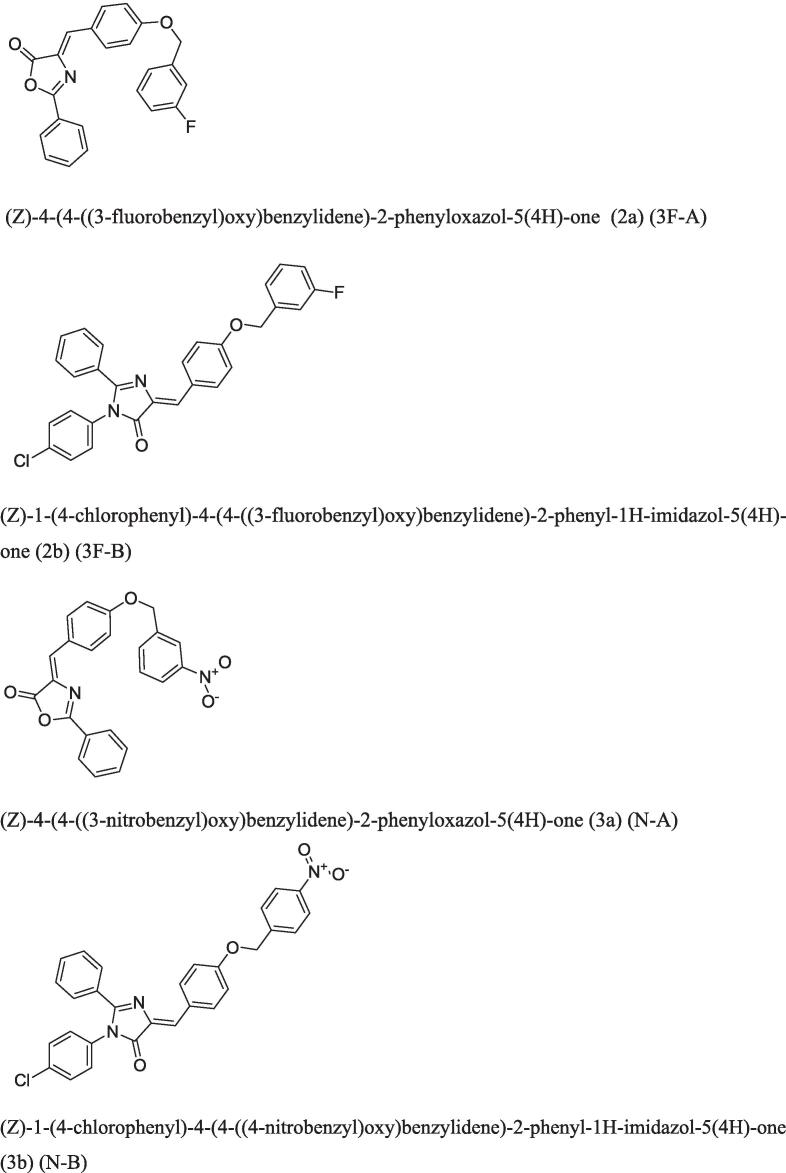
Structure of selective new oxazolones (3F-A, 3F-B) and imidazolones (N-A, N-B) for *in vivo* pharmacological investigations.

Intermediates 1 (a-b) i-e 4-((3-flurobenzyl)oxy)benzaldehyde, 4-((4-nitrobenzyl)oxy) benzaldehyde were synthesized by using substituted benzaldehyde with benzyl halides which further reacted with hippuric acid to yield oxazolones 2 (a-b) which then reacted with 4-chloroaniline yielded corresponding imidazolines 3 (a-b) (Fig. 1).

### 2.3 Molecular docking

Protein structure was downloaded from the protein data bank. https://www.rcsb.org/home/home.do Protein was cleaned and ligands were drawn in Discovery studio visualizer and saved in pdb format. All Pdb files were converted to Pdbqt format by AutoDock tools. The docking was performed using AutoDock Vina 1.2.3. The scoring function was selected as Vina, exhaustiveness was set at 25. The active site of the catalytic domain was identified with the help of the location of co-crystallized COX-2 and TACE (TNF-α converting enzyme). The structure of indomethacin was also docked to give a reference value of binding affinity and to validate the procedure by generation of a similar pose/conformation as that of co-crystallized indomethacin (Ansari et al., 2019).

### 2.4 Animals

Albino mice of either sex weight 20–25 g were used and obtained from the animal house of the University of the Punjab, Lahore Pakistan, and kept in temperature temperature-controlled environment (20–25 °C). Standard rodent feed and water *ad libitum* were provided. All experiments were approved by the Institutional Ethics Review Board, University of the Punjab, Lahore Pakistan (Ref. no. 35/FIMS). The study was carried out in agreement with the guidelines of “Principals of Laboratory Animal Care”.

### 2.5 Carrageenan-induced paw edema

The anti-inflammatory activity of newly synthesized compounds was evaluated by the carrageenan-induced paw edema model described by Winter and his coworkers in 1962 (Asadi et al., 2021). The albino mice of both genders with a weight range of 20–25 g were kept under standard laboratory conditions for one week in the animal house of the Punjab University College of Pharmacy, University of Punjab, Lahore, Pakistan to acclimatize the animals and to decrease the stress level. Animals were weighed and randomized into 7 groups (n = 5) including the control group (normal saline), carrageenan-induced diseased group (0.5 % 10 mg/kg), reference group (indomethacin 10 mg/kg), and treatment groups (10 mg/kg) of each newly synthesized compounds (3F-A, 3F-B, N-A, N-B). The new compounds were administered intraperitoneally. The control group received only normal saline, and the reference group or positive control received indomethacin at 10 mg/kg dose. After 60 min of dose administration, 0.1 ml of 0.5 % carrageenan was injected into sub plantar region of the left hind paw of each mouse. The paw volume was measured by using a mercury phlethysmometer immediately, before, and on an hourly basis for up to 5 h. After 6 h the mice were sacrificed and paw tissues were collected.

### 2.6 Tissue collection

After 6 h of injecting carrageenan the paw samples were collected by euthanizing the mice by cervical dislocation. The paw samples were divided into two parts, one stored at −80 °C for molecular analysis, while the other preserved in 10 % formalin for histopathological evaluation. The samples (50 g) were homogenized with PBS (PH 7.4) and centrifuged at 2500 rpm for half an hour followed by the collection of supernatant which was then used for molecular analysis.

### 2.7 Determination of oxidative stress markers

The tissue antioxidant effect of new compounds (3F-A, 3F-B, N-A, N-B) was analyzed in homogenized paw tissue of mice by measuring the levels of glutathione (GSH), catalase, glutathione s-transferase (GST) and lipid peroxidation (LPO) by previously mentioned protocol (Badshah et al., 2023). Results were expressed as µmol /mg of protein for GSH, µmol of CDNB conjugate/min/mg of protein for GST, µmol of H2O2/minute/mg of protein for catalase, and nmol/min/mg of protein for lipid peroxidation.

### 2.8 Hematoxylin and eosin (H & E) staining

Five mice were used in each group for morphological analysis. 10 % paraformaldehyde was used for mice tissue fixation and would be embedded in paraffin, until further analysis. The tissues were divided into 5 mm sections using a rotary microtome, and then they were stained with H & E (Hematoxylin and Eosin). With the help of an optical microscope, the paw tissues were examined and images were taken (Aslam et al., 2022).

### 2.9 Enzyme-Linked Immunosorbent Assay (ELISA)

Inflammatory markers COX-2 and TNF-α were detected in the paw tissues of mice following the instructions of the ELISA kit manufacturer (Elabscience). The tissues were kept at −80 °C in a bio-freezer for storage. The tissue samples were homogenized with the help of Silent Crusher M (heidolf) at 15 rpm × 1000 followed by the collection of supernatant after 1 h of centrifugation at 1,350*g* for 60 min. The supernatant was analyzed for COX-2 ELIZA kit (Cat No. PRS.30205Ra) and TNF-α ELIZA kit (Cat No. PRS-30651Ra) (Azmatullah et al., 2022).

### 2.10 Real-time- polymerase chain reaction

Paw tissue of mice (n = 3) after homogenization was exploited to extract ribonucleic acid (RNA) by triazole method according to the following procedure described by the manufacturer of RNA extraction kit (SYBR Select PCR Master Mix Kit).cDNA was prepared by reverse transcriptase enzyme using 1–2 µg of total RNA. The amplification of cDNA was achieved by real-time time-PCR (RT-PCR) using a thermocycler. The expression of mRNA was normalized to the expression level of GAPDH. The determination of relative gene expression was carried out by the 2^^ΔΔ-ct^ method for real-time quantitative PCR primer sequence for COX-2 and GAPDH as follows:

CAAGCAGTGGCAAAGGCCTCCA (COX-2 forward).
GGCACTTGCATTGATGGTGGCT (COX-2 reverse).
CAACTCCCTCAAGATTGTCAGCAA (GAPDH forward).
GGCATGGACTGTGGTCATGA (GAPDH reverse).

## 3 Results

### 3.1 Molecular docking

**Fig. 2:**
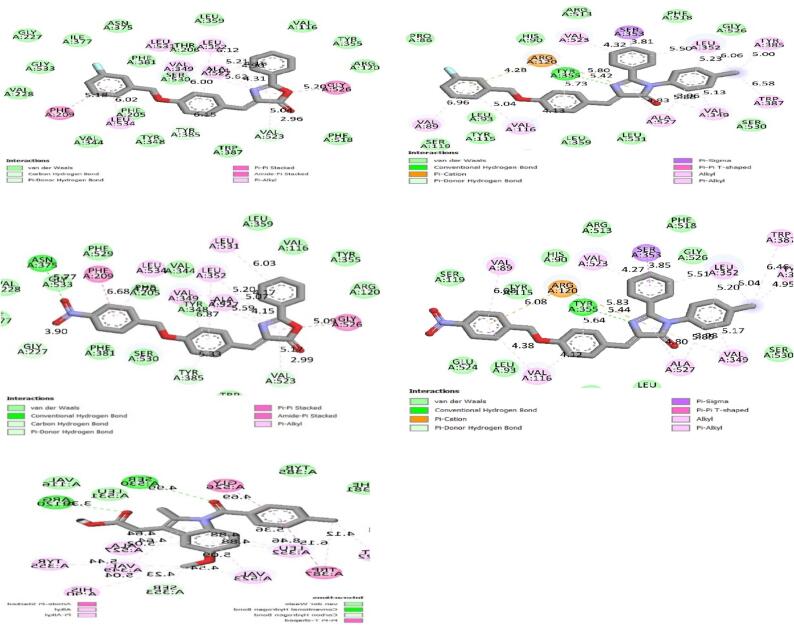
2D images of new compounds 3F-A, 3F-B, N-A and N-B showing binding affinities for COX-2 receptor in comparison with indomethacin. Vander Waals interactions, conventional hydrogen bonds, pi-pi, and pi-sigma bonding show a prominent role in receptor binding.

**Fig. 3:**
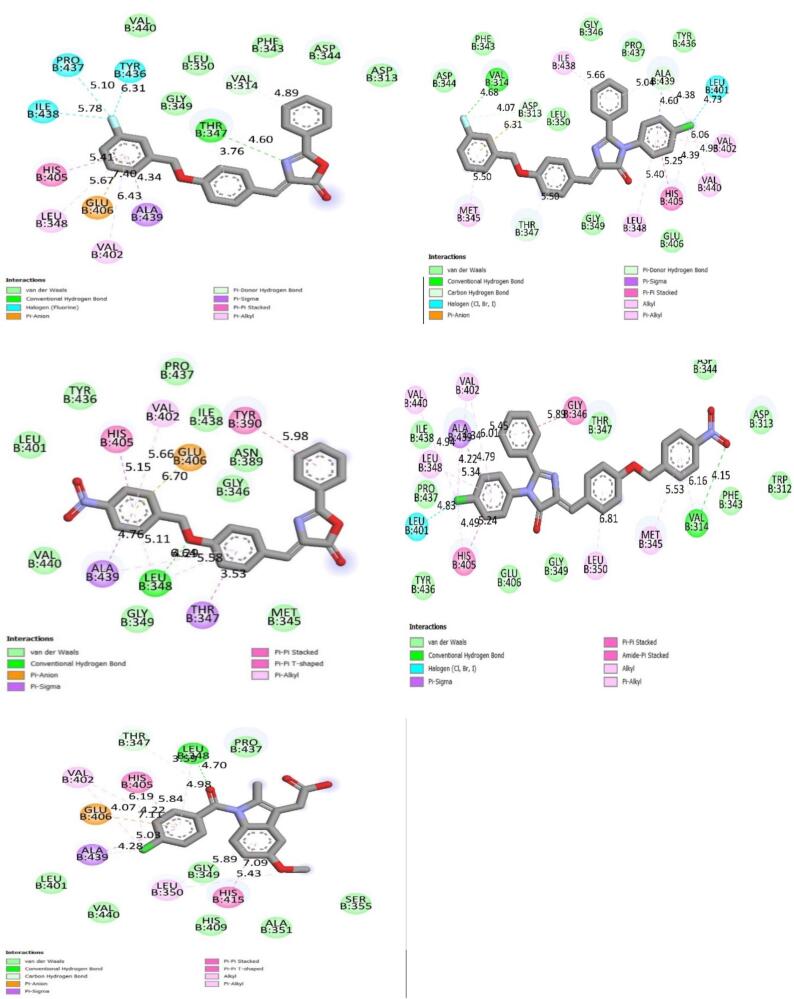
2D images of new compounds 3F-A, 3F-B, N-A, and N-B showing binding affinities for TACE receptors in comparison with indomethacin. Vander Waals interactions, conventional hydrogen bonds, pi-pi, and pi-sigma bonding show significant roles in receptor binding.

Docking studies of the compounds against targeted protein cox-2 and TACE manifested good binding affinities for both the targets supporting their anti-inflammatory potential. 2D images of docked Compounds (3F-B belonged to imidazolones and N-A belonged to oxazolones) against COX-2 and 2D images of docked compound (3F-A belonged to oxazolones and N-B belonged to imidazolones) against TACE are shown in Fig. 2, Fig. 3 respectively and compared with docked indomethacin (standard drug). Results are expressed concerning estimated free binding energies (docking score) in Table 1. 3F-B showed maximum binding affinity for COX-2 while N-B exhibited maximum binding affinity for TACE as indicated by their minimum free binding energy (−9.736 kcal/mol and −9.860 kcal/mol respectively).

**Table 1:** Estimated free energy of binding of synthesized compounds on the target COX-2 and TACE1.

Protein	5ikr (COX-2)		2ddf (TACE)
S. No.	Ligand	Affinity (kcal/mol)	Affinity (kcal/mol)
1	3F-A	−8.687	−7.995
2	3F-B	−9.736	−8.726
7	N-A	−8.488	−8.203
8	N-B	−7.629	−9.860
13	Indomethacin	−6.597	−7.570

### 3.2 Carrageenan-induced paw edema

**Fig. 4:**
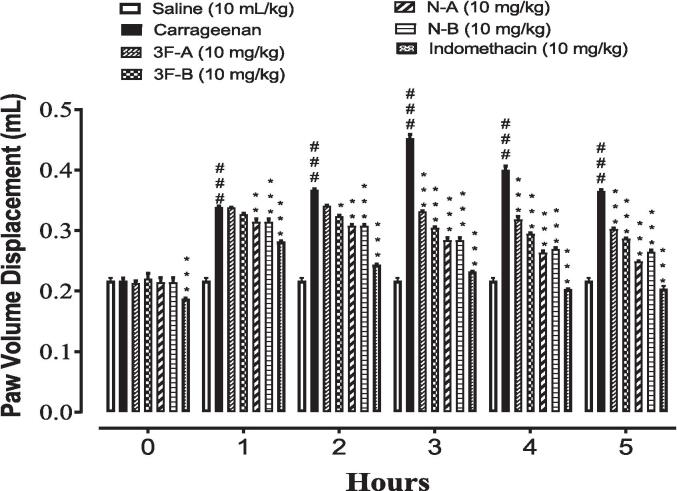
Effect of synthesized compounds (3F-A, 3F-B, N-A, N-B) and standard drug indomethacin vs. carrageenan-induced paw edema. Values expressed as mean ± SEM (n = 5). One-way ANOVA with post hoc Tukey’s test. ^###^*P* < 0.001 vs. saline,*P < 0.05, ^**^P < 0.01, ^***^P < 0.001 vs. carrageenan group.

Sub-plantar administration of carrageenan in rats culminated in a time-dependent increase in paw volume (Fig. 4). However, the synthesized compounds 3F-A, 3F-B, N-A, and N-B decreased the paw edema in a dose-dependent manner starting from zero to 5 h after an injection of carrageenan. All compounds at 10 mg/kg dose depicted significant reduction in inflammation with *p < 0.05, **p < 0.01, ***p < 0.001 as compared to the Carrageenan group. Among all, compounds, NA was found to be the most effective compound as it evinced the maximum reduction in inflammation (***p < 0.001 as compared to the Carrageenan group).

### 3.3 Effect on oxidative stress markers

**Fig. 5:**
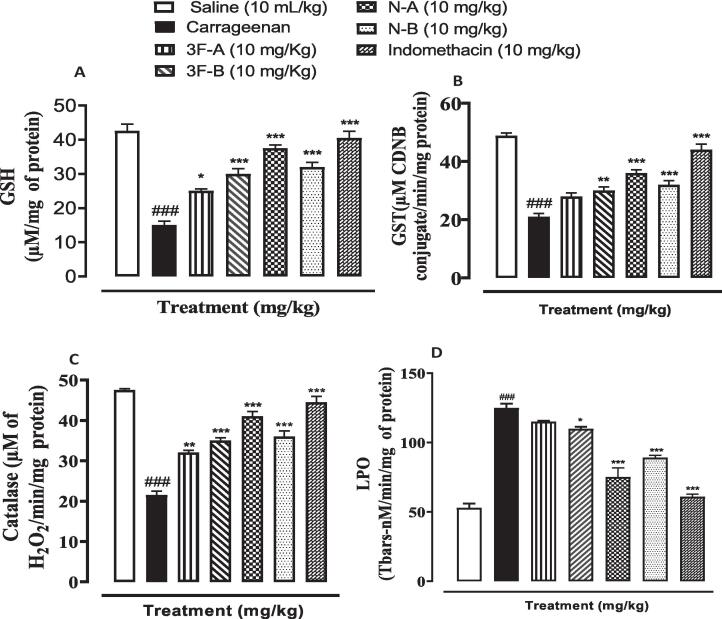
Effect of synthesized compounds (3F-A, 3F-B, N-A, N-B) and standard indomethacin drug vs. (a) glutathione s- transferase (GST), (b) reduced glutathione (GSH), (c) Catalase and (d) Lipid peroxidation (LPO) in carrageenan-induced paw edema in paw tissues of mice. Values expressed as mean ± SEM (n = 3). One-way ANOVA with post-hoc Tukey’s test. ^###^*P* < 0.001 vs. saline,*P < 0.05, ^**^P < 0.01, ^***^P < 0.001 vs. carrageenan group.

To evaluate the antioxidant activity of synthesized compounds the *in vivo* antioxidant tissue activity was performed by accessing the level of catalase, GSH, GST, and LPO on paw tissues. The results indicated that new compounds improved oxidative stress and restored the expression of enzymes. GSH, GST, and catalase in the diseased group (Carrageenan) were reduced while LPO was increased (^###^*P* < 0.001 vs. saline). Treatment with new compounds significantly increased the levels of catalase, GSH, and GST in paw tissues while LPO was decreased (*P < 0.05, ^**^P < 0.01, ^***^P < 0.001 vs. carrageenan group) (Fig. 5).

### 3.4 Hematoxylin and eosin (H & E) staining

**Fig. 6:**
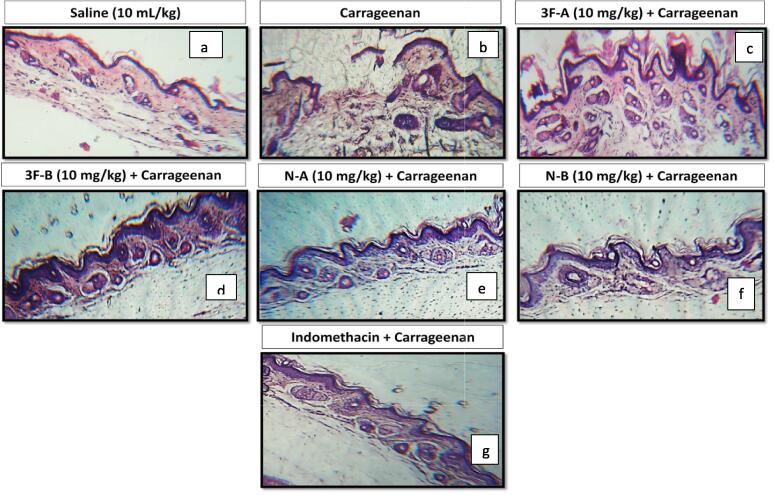
Histopathological slides showing the morphological action of synthesized compounds and indomethacin on carrageenan-induced paw edema using H&E staining procedures in mice paw tissues.

The histopathological changes in paw tissue by H&E staining revealed intact structures without any damage with normal appearance of tissues in saline group Fig. 6(a) while abnormal cellular morphology with disruption of cellular entities indicating tissue damage by induction of inflammation caused by carrageenan was observed in diseased group Fig. 6(b). However, treatment groups shown in Fig. 6(c)–(f) (10 mg/kg) and standard indomethacin (10 mg/kg) shown in Fig. 6(g) exhibited a significant improvement in the cellular structures. Maximum improvement in the morphology of tissue was observed by compound N-A as evident in Fig. 6(e) indicating the preservation of normal structure and function of tissue.

### 3.5 ELISA

**Fig. 7:**
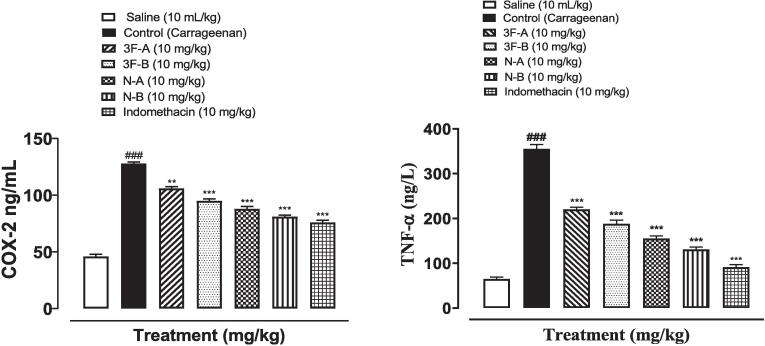
Effect of tested compounds (3F-A, 3F-B, N-A, N-B) and indomethacin against COX-2 and TNF-α levels in carrageenan-induced paw edema in mice paw tissues using ELISA technique. Values expressed as mean ± SEM (n = 3). One-way ANOVA with post-hoc Tukey’s test. ^###^*P* < 0.001 vs. saline, ^**^P < 0.01, ^***^P < 0.001 vs. carrageenan group.

The expression of inflammatory markers COX-2 and TNF- α were measured by Enzyme-linked immunosorbent assay (ELISA) that indicated increased expression of these markers in the diseased group (^###^*P* < 0.001 vs. saline), while a notable reduction in expression of these markers occurred in treatment groups and the standard group that revealed a promising anti-inflammatory effect of newly synthesized compounds (^**^P < 0.01, ^***^P < 0.001 vs. carrageenan group). Compound N-B showed maximum reduction in protein expression followed by N-A, F-B, and F-A as shown in Fig. 7.

### 3.6 Quantification of mRNA levels

**Fig. 8:**
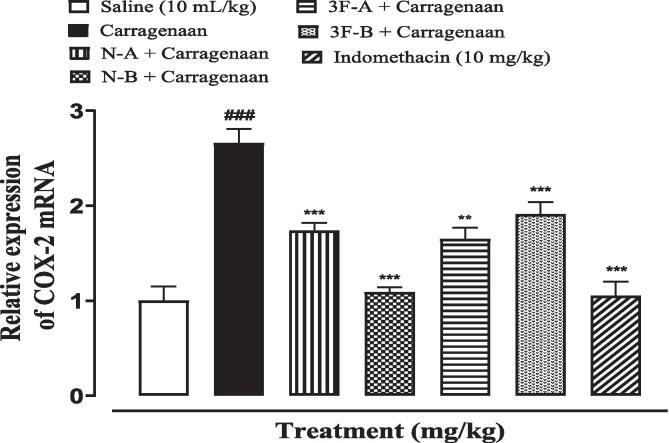
Inhibitory effect of newly synthesized compounds and indomethacin against mRNA of COX-2 expression in carrageenan-induced paw edema in mice paw tissues using RT-qPCR technique. Values expressed as mean ± SEM (n = 3). One-way ANOVA with post-hoc Tukey’s test. ^###^P < 0.001 vs. Saline group and ^**^p < 0.001, ^***^p < 0.001 vs. carrageenan group.

Real-time PCR determined the fold of expression of COX-2 mRNA in carrageenan-induced paw edema. COX-2 mRNA levels of expression were increased in the carrageenan group (^###^*P* < 0.001 vs. saline). The test compounds and indomethacin-treated groups exhibited an enormous decrease in the expression of COX-2 mRNA levels (^**^P < 0.01, ^***^P < 0.001 vs. carrageenan group) (Fig. 8).

## 4 Discussion

Inflammation is a protective strategy against harmful events such as tissue injury, microbial infections, and other pernicious conditions. Different types of inflammation are initiated by numerous stimuli and are accomplished by diverse regulatory mechanisms. Natural sources especially medicinal plants continue to be an impactful source of natural products for the treatment of various health conditions. A plethora of research related to the anti-inflammatory potentials of diverse plant families like Euphorbiaceae (*Acalypha indica* L., *Phyllanthus niruri* L.), Acanthaceae (*Ruellia asperula),* Amaranthaceae*, (Alternanthera brasiliana* (L.), Apocynaceae (*Himatanthus drasticus (Mart.*), Boraginaceae, (*Momordica charantia* L.). Solanaceae, (*Solanum paniculatum* L.), etc. have been investigated in recent times (Khan et al., 2022).

Apart from natural sources, chemical synthesis has played a colossal role in the development of very effective new therapeutically active compounds (Noman et al., 2022). Various heterocyclic ring-containing compounds like triazine, benzimidazole (Al-Mulla, 2017) thiadiazole, oxodiazole (Razzaq et al., 2022), pyrazole, pyrimidine, indole (Qazi et al., 2022), thiazole, oxazole, and imidazole derivatives (Nunes et al., 2020) have been exposed for their anti-inflammatory and anti-oxidant potentials and proved to be very effective therapeutic agents because of the boundless efforts in the field of synthesis.

Aromatic aldehydes, halides, and anilines have been used in the synthesis of oxazole and Imidazoles. Various schemes for the synthesis of oxazoles and imidazoles have been reviewed in the literature and a new series has been prepared and reported in the literature (Saleem Naz Babari et al., 2024) following the schemes given by Kumar and his coworkers for oxazole and EL-Araby and his coworkers for imidazole with minor chemical modifications (Abdellatif and Fadaly, 2017, Zheng et al., 2020). The newly synthesized compounds were then investigated for anti-inflammatory effects by carrageenan-induced paw edema model. The inflammatory response was quantified by an increase in paw size (edema) which was maximal at 5 h after carrageenan injection (Ahmed, 2011). *In silico* studies of Compounds were then performed to understand the binding affinity and binding patterns of newly synthesized compounds with COX-2 and TACE receptor proteins that showed Vander Waals, conventional hydrogen bonds, pi-sigma bonds, pi-pi, pi- alkyl type interactions between ligands and targeted receptors. In compound 3F-B VAL: 116, VAL: 89, VAL: 523, SER: 353, and LEU: 353 are involved in binding with aromatic rings while ARG: 120, TYR: 355, ALA: 527 were found to be involved in binding with imidazole ring, ARG: 120, VAL: 89, VAL: 116 exhibited the affinity for fluro benzyl ring. In compound N-A LEU: 531, TYR: 348, PHE: 209, and PHE: 205 appeared to be interacting with aromatic rings, GLY: 526, LEU: 352, VAL: 523 and were thus involved in binding with oxazole ring, GLY: 77 AND ASN: 375 participated in binding with nitro group of phenyl moiety. Similarly, compound 3F-A was found to be involved in the interactions of various amino acid residues of receptors like VAL: 314, ALA: 439, GLU: 406, HIS: 405 and exhibited an affinity for aromatic rings of compound. TYR: 436, PRO: 437, and ILE: 438 showed interactions with the fluorine atom of the fluro benzyl group, and THR: 347 showed a direct interaction with the nitrogen of the oxazole ring. Aromatic rings of Compound N-B depicted interaction with GLY: 346, LEU: 350, ALA: 439, LEU: 348, and HIS: 405 while the nitro group was involved in binding with VAL: 314. LEU: 401 showed great affinity for the chlorine atom of chlorophenyl moiety directly attached to the imidazole ring. The results obtained from molecular docking indicate that out of the two series the compound (Z)-4-(4-((3-nitrobenzyl)oxy)benzylidene)-2-phenyloxazol-5(4H)-one (N-A) belongs to oxazole series having nitro functional group exhibits significant binding affinity for a targeted receptor that supports the results of anti-inflammatory activity by carrageenan-induced paw edema model where the same compound from aforementioned series was found to be most effective because the nitro group is directly involved in the binding of amino acid residues of receptor proteins.

Similarly, compound (Z)-1-(4-chlorophenyl)-4-(4-((4-nitrobenzyl)oxy)benzylidene)-2-phenyl-1H-imidazol-5(4H)-one (N-B) from imidazole series also showed significant binding fashion with the target protein, indicates that the compound has a more favorable binding pattern with the receptors. The results of ELIZA and PCR also indicate the maximum reduction in expression of COX-2 and TNF- α shown by the same compound. The results support that in the present study the nitro compounds no matter belongs to which series were found to be more active in the inhibition of inflammation than fluro compounds because the nitro group directly participates in the binding of receptor proteins than the fluro groups.

Like the previously reported oxazole and imidazole derivatives having anti-inflammatory potentials (Garg et al., 2023, Shakya et al., 2016, Puratchikody and Doble, 2007, Husain et al., 2009). The newly synthesized compounds were also exploited for anti-inflammatory potential by carrageenan-induced paw edema model and results were compared with standard anti-inflammatory drug (indomethacin 10 mg/kg). The results exhibited a pronounced response of each new compound on reduction of paw volume displacement up to 5 h indicating a gradual decrease in inflammation or edema in the paw of mice. These new derivatives having different substituents at different positions like the earlier reported compounds synthesized from aforementioned starting materials showed significant effects in reducing the inflammation and are comparable with the anti-inflammatory effects of previous derivatives investigated for *in vivo* anti-inflammatory effect (El-Araby et al., 2012).

Furthermore, these findings support the previously mentioned results present in literature that oxazole and imidazole ring having halogen groups (fluorine, chlorine) and nitrogen groups in their structures displayed significant COX-2 inhibitory potentials (Abdellatif and Fadaly, 2017, Kalgutkar, 1999). Similarly, literature also described the fact that un-substituted imidazoles were found completely inactive as COX-2 inhibitors but the addition of halo substituents on 1,5 diaryl imidazole made them effective COX-2 inhibitors (Che et al., 2010). All compounds belonging to both oxazole and imidazole series were effectively reducing the inflammation supporting the previous concept present in literature that both halo and nitro groups when attached to basic benzylidene moieties of both oxazole and imidazole have played a key role in the suppression of inflammatory markers (Joshi et al., 2017, Ranjith, 2016).

In recent years, evidence has emerged that oxidative stress plays a pivotal role in the development of inflammation (Hussain et al., 2016) and thus has an important role in the pathophysiology of several illnesses, such as cardiovascular diseases (Dubois-Deruy et al., 2020), diabetes (Piconi et al., 2003), cancer (Hayes et al., 2020) or neurodegenerative diseases (Chen et al., 2012). All phases of inflammation including endogenous hazardous signals produced by damaged tissues, their recognition by innate immune receptors, and initiation of signaling pathways by adaptive cellular response to such signals are affected by oxidants (Nunes et al., 2020).

The level of catalase reduced glutathione, and glutathione s-s-transferase along with lipid peroxidation has been assessed to evaluate the tissue antioxidant effect of various compounds having anti-inflammatory potential (Alblihed, 2020) because oxidative stress and inflammation are interlinked phenomena. Oxidative stress exaggerates the process of inflammation (McGarry et al., 2018). Literature has shown the oxazole and imidazole derivatives were studied for *in vitro* antioxidant effects where they remarkably exhibited their antioxidant potentials (Mathew et al., 2013). The new compounds were also studied for tissue antioxidant effects and results were compared with indomethacin as a standard drug. The results specified that compound N-A belonged to the oxazole series and showed impressive restoration of the aforementioned enzymes and reduction in LPO compared to others. Similarly, enhanced reduction in lipid peroxidation has been observed by the same compound. These results can also be related to *the in vitro* antioxidant effect of oxazole present in the literature showing oxazole rings having nitro and fluro substitution on terminal aryl ring displayed maximum antioxidant effect (Yatam et al., 2018).

Literature has shown histopathological studies of various synthetic derivatives to understand how these derivatives produce their effect in the restoration of inflamed tissue morphology by comparing with the standard drug indomethacin (Kumar et al., 2008, Hussein et al., 2013, Amir et al., 2008, Rana et al., 2008). Similarly, new targeted compounds were investigated for their effects on the morphology of carrageenan-induced inflamed paw tissues by the H & E staining technique. The damaged cellular boundaries and raptured cellular organelles were seen to be restored in the treatment group tissue compared to the diseased group tissue. Again, compound N-A having oxazole ring with nitro benzyl moiety showed maximum observable retrieval of cellular morphology stipulating a good anti-inflammatory effect of this compound justifying our previously mentioned result of tissue antioxidant effect of the same compound.

The enzyme-linked Immunosorbent assay indicated the suppressive effect of new compounds on the overly expressed inflammatory markers COX-2 and TNF- α that were comparable to the standard indomethacin drug. The maximum, reduction in enzyme expression was shown by compound N-B belongs to imidazole having nitro phenyl group suggesting its superior effect as compared to other compounds. This result is in concordance with those already present in the literature that imidazole ring has shown tremendous effects on COX-2 suppression (Lamie et al., 2018, Reddy et al., 1994).

PCR was done to elucidate the inflammatory pathway and to identify the role of newly synthesized compounds in inhibiting the proposed mechanism of inflammation. In literature synthetic compounds were being evaluated for prevention of inflammation by suppressing inflammatory mediators like TNF-α, COX-2, IL-1β, and IL17 (Zubair et al., 2022). Keeping the literature survey in mind the newly synthesized compounds were being exposed for suppression of mRNA level of inflammatory mediators, particularly for COX-2 and the results indicated a successful suppression of COX-2 mRNA level that was comparable to the standard anti-inflammatory drug (indomethacin). All compounds reduced the expression of the mRNA level of COX-2 but compound N-B was found most remarkable in this regard indicating that the incorporation of the nitro group in the basic imidazole ring may enhanced the suppression of the aforementioned inflammatory marker.

## 5 Conclusion

We investigate new oxazole and imidazole derivatives for their anti-inflammatory potential as COX-2 inhibitors. The evidence collected from the present study highlights that the presence of halo and nitro groups in both the basic oxazole and imidazole moieties displays a prominent effect as COX-2 inhibitors. All the studies performed here describe the successful effect of these new candidates in reducing the expression of inflammatory mediators. The reduction in the expressions of inflammatory mediators especially COX-2 suggests that it can be the most likely mechanism of action of new compounds as an anti-inflammatory agent. Future investigations can be carried out to authenticate their mechanism of action by performing further *in vivo* studies. Furthermore, SAR studies can also be carried out to understand how new electron-withdrawing or electron-donating group substitution in basic structure skeleton can affect biological activity.

## CRediT authorship contribution statement

**Iqra Saleem Naz Babari:** Writing – original draft, Software, Investigation, Data curation. **Muhammad Islam:** Writing – review & editing, Supervision, Resources, Project administration, Methodology, Conceptualization. **Hamid Saeed:** Writing – original draft, Software, Resources, Investigation, Formal analysis. **Humaira Nadeem:** Writing – review & editing, Visualization, Supervision, Project administration, Formal analysis. **Hassaan Anwer Rathore:** Writing – review & editing, Supervision, Resources, Methodology, Conceptualization.

## Declaration of competing interest

The authors declare that they have no known competing financial interests or personal relationships that could have appeared to influence the work reported in this paper.
